# Increased risk of poor clinical outcome in COVID‐19 patients with diabetes mellitus and in‐hospital mortality predictors: A retrospective cohort from a tertiary hospital in Indonesia

**DOI:** 10.1002/edm2.454

**Published:** 2023-10-09

**Authors:** Md Ikhsan Mokoagow, Dante Saksono Harbuwono, Ida Ayu Kshanti, C. Martin Rumende, Imam Subekti, Kuntjoro Harimurti, Khie Chen, Hamzah Shatri

**Affiliations:** ^1^ Division of Endocrinology, Metabolism, and Diabetes Department of Internal Medicine Fatmawati General Hospital Jakarta Indonesia; ^2^ Department of Internal Medicine, Faculty of Medicine Universitas Indonesia Jakarta Indonesia; ^3^ Division of Endocrinology and Metabolism Department of Internal Medicine, Faculty of Medicine Dr. Cipto Mangunkusumo National Referral Hospital, Universitas Indonesia Jakarta Indonesia; ^4^ Clinical Epidemiology Unit Department of Internal Medicine, Faculty of Medicine Dr. Cipto Mangunkusumo National Referral Hospital, Universitas Indonesia Jakarta Indonesia

**Keywords:** COVID‐19, diabetes mellitus, intensive care, mortality, predictor

## Abstract

**Aim:**

To determine association between diabetes in confirmed cases of COVID‐19 and intensive care admission and in‐hospital mortality, evaluate several laboratory parameters as mortality predictor and develop predictors of in‐hospital mortality among diabetics with COVID‐19.

**Methods:**

This retrospective cohort recruited all cases of COVID‐19 hospitalized in Fatmawati General Hospital from March to October 2020. Inclusion criterion was RT‐PCR confirmed cases of COVID‐19 who aged 18 years and older while exclusion criteria were incomplete medical record or cannot be found and pregnant women.

**Results:**

We enrolled 506 participants to this study with median age of 51 years (IQR:22), female (56.32%), and diabetes (28.46%). Diabetes increased intensive care admission (adjusted OR: 2.57; 95% CI: 3.52–10.43) and in‐hospital mortality (adjusted OR: 2.50; 95% CI: 1.61–3.89). In predicting in‐hospital mortality, ferritin and lactate dehydrogenase offered an acceptable discrimination, AUC: 0.71 (95% CI: 0.62–0.79) and AUC: 0.70 (95% CI: 0.61–0.78), respectively. The optimal cut‐off of predicting mortality for ferritin was 786 g/mL and for LDH was 514.94 u/L. Factors include age above 70 years old, RBGs level on admission above 250 mg/dL or below 140 mg/dL, ferritin level above 786 ng/mL and presence of ARDS increased the odds of mortality among individuals with diabetes.

**Conclusions:**

Diabetes increases risk intensive care admission and in hospital mortality in COVID‐19. Multivariate analysis showed that older age, RBG on admission, high ferritin level, presence of ARDS increased the odds of mortality among individuals with diabetes.

## Graphical abstract

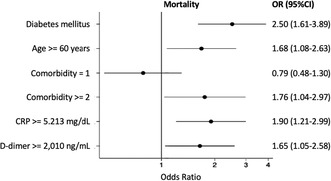

## 1 INTRODUCTION

Coronavirus disease‐2019 (COVID‐19) first identified in Wuhan, China and subsequently spread worldwide hence emerged as global health problem. Currently its case fatality rate in Indonesia exceeded the global rate.^1^ Presence of comorbidity contribute to poor clinical outcome.^2^ COVID‐19 patients with diabetes are at risk of developing poor outcome during hospitalization.^3^, ^4^ Our preliminary data in early pandemic documented half of individuals with diabetes admitted to our hospital due to COVID‐19 were in critical diseases with a mortality rate of over 60% in this group.^5^ Identifying individuals with diabetes who are at higher risk of having clinical deterioration is of clinical importance.

Laboratory findings as predictors for poor outcome in COVID‐19 has been extensively studied. Parameters such as C‐reactive protein, lactate dehydrogenase, ferritin, D‐dimer, neutrophils to lymphocyte ratio and monocyte to lymphocyte ratio have been associated with poor clinical outcome in COVID‐19.^6^, ^7^, ^8^, ^9^, ^10^, ^11^, ^12^, ^13^, ^14^, ^15^, ^16^, ^17^

It is conceivable that diabetes may contribute to poor clinical outcome and laboratory findings may predict in hospital mortality. Nevertheless, studies relating these occurrence remains limited and laboratory finding for predicting worse outcome in DM with COVID‐19 may provide alternative insight in different populations. We sought to determine the association between diabetes in confirmed cases of COVID‐19 and intensive care admission and in‐hospital mortality, evaluate several laboratory parameters as mortality predictor, and develop predictors of in‐hospital mortality among diabetics with COVID‐19.

## 2 METHODS

### 2.1 Study design and participant

This is a single‐centre, retrospective cohort study conducted in Fatmawati General Hospital from March to October 2020. Fatmawati General Hospital is a tertiary hospital located in South Jakarta. This hospital served as a referral hospital for severe COVID‐19 for three consecutive cities, including South Jakarta, Depok and South Tangerang cities (total population of 6,343,000). A total of 1872 COVID‐19 patients (confirmed and probable) were admitted during the study period. We included all COVID‐19 confirmed individuals diagnosed based on RT‐PCR test of nasal or oropharyngeal swab specimen, aged above 18 years or older and at least had one measurement of random blood glucose test during admission. Individuals who were pregnant and data for the outcome cannot be achieved excluded from this study.

We reviewed demographical data, comorbidities and laboratory findings based on patients' medical charts. Diabetes was determined based on the patient's medical history and laboratory parameters, including random blood glucose ≥200 mg/dL in two consecutive measurement while hospitalized in COVID‐19 isolation ward, fasting blood glucose ≥126 mg/dL or HbA1C ≥ 6.5. Comorbidity was determined based on the patient's medical history and categorized into without comorbidity, presence of one comorbidity and presence of two or more comorbidity.

### 2.2 Laboratory examinations

Laboratory findings consisted of random blood glucose test (RBGs), C‐reactive protein (CRP), Ferritin, D‐dimer, lactate dehydrogenase (LDH), neutrophile to lymphocyte ratio (NLR) and monocyte to lymphocyte ratio (MLR), all obtained at the time of admission. The disease severity was categorized based on WHO COVID‐19 severity and assessed by the attending physician.

According to the WHO disease severity, mild disease is defined by the care definition for COVID‐19 without evidence of viral pneumonia or hypoxia, moderate is defined by clinical sign of pneumonia but no sign of severe pneumonia, severe pneumonia is defined by clinical signs of pneumonia plus one of the following: respiratory rate > 30 breath/min; severe respiratory distress; spO_2_ < 90% room air, critical illness is grouped into ARDS, sepsis and septic shock.

Acute respiratory distress syndrome (ARDS) was determined based on oxygen saturation measured by pulse oximetry/FiO_2_ ratio < 315 at the time of admission. We assessed the incidence of intensive care treatment, and mortality of COVID‐19 patients with or without prior diabetes condition treated in our hospital.

Laboratory data cut‐off was obtained based on previously published research for predicting critical conditions in COVID‐19 patients. The cut‐off for laboratory findings were RBG 180 mg/dL,^18^ CRP 2.587 mg/dL,^14^ ferritin 163.5 ng/mL,^8^ d‐dimer 565 ng/mL,^8^ LDH 277 u/L,^16^ NLR 5.87,^19^ MLR 0.30,^14^ respectively for intensive care outcome, and RBG 180 mg/dL,^18^ CRP 5.213 mg/dL,^13^ ferritin 304.3 ng/mL,^17^ d‐dimer 2010 ng/mL,^9^ LDH 353.5 u/L,^15^ NLR 8.085,^14^ MLR 0.364,^20^ respectively for mortality outcome. (Table S1).

### 2.3 Outcome

The outcome of the study was the incidence of intensive care treatment and mortality of COVID‐19 patients with or without prior diabetes conditions treated in our hospital.

### 2.4 Statistical analysis

Numeric data were presented as a median and interquartile range (IQR) or mean and standard deviation (SD) as determined by the Shapiro–Wilk test. Categorical data were presented as frequency and percent. Bivariate analysis using the chi‐squared test and multivariate logistics regression was performed to obtain crude and adjusted odds ratios. We assessed the association between diabetes and outcome using causative models. Nearly all study variables were assessed for their relationship with the outcomes. Backward selection for selecting covariates as confounding factors was used. Suppose the exclusion of covariate changes the OR crude of diabetes by more than 10% than the covariates is considered a potential confounder. If there is a clinically meaningful relationship, the potential confounders are regarded as confounders and adjusted ORs of association between diabetes and outcomes were obtained. We measured the performance of laboratory findings by measuring the area under curve, sensitivity and specificity. The optimal cut‐off value is measured by determining the closest distance between the point (0.1) and the point on the ROC curve (d) and determining the farthest vertical distance between the line of equality and the point on the ROC curve (Youden index). All the analysis was performed using STATA Version 12. The study was reviewed and approved by the Research Ethic Committee of Fatmawati General Hospital (27/KEP/XII/2020).

## 3 RESULTS

### 3.1 Patients characteristics

**FIGURE 1:**
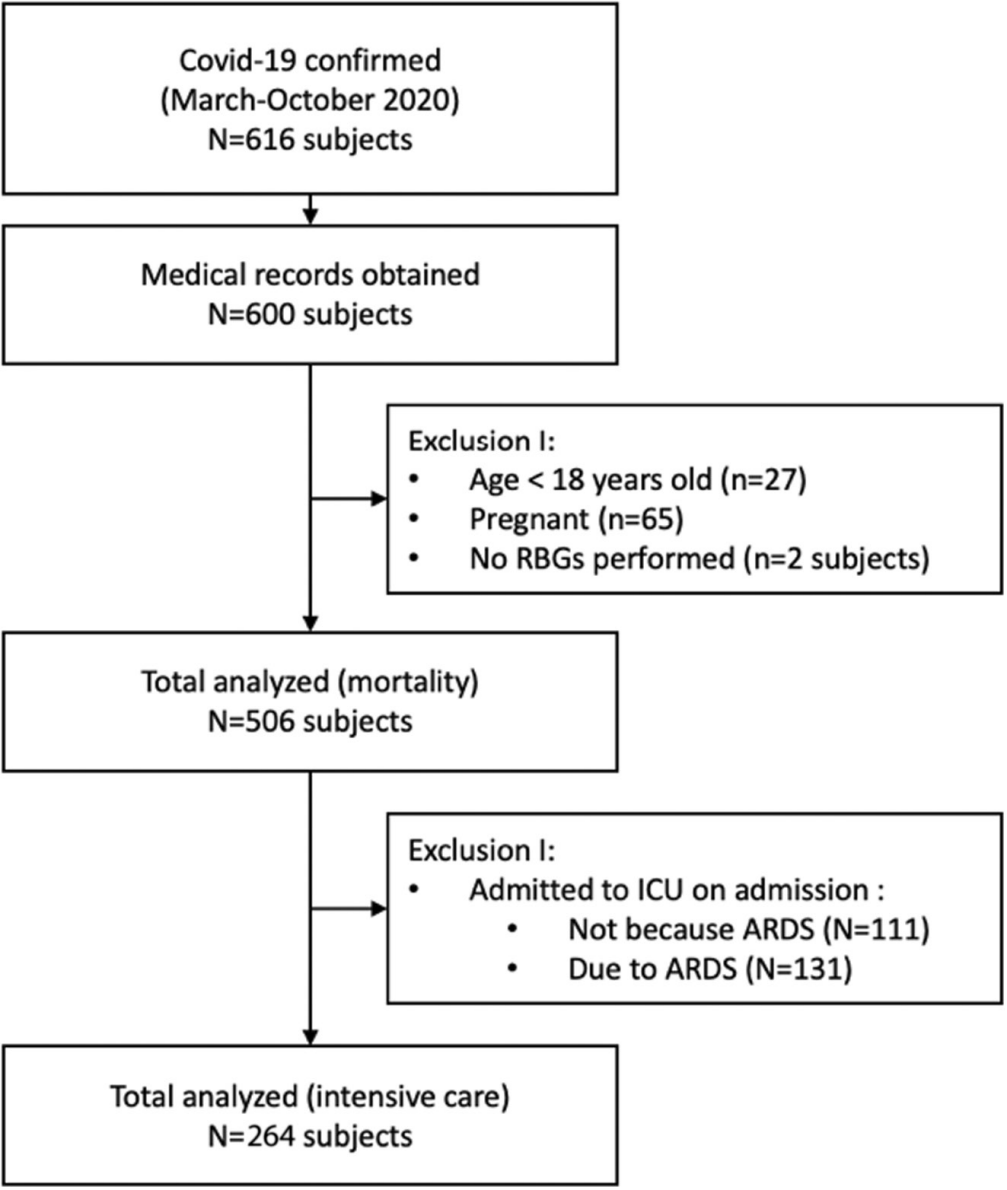
Flow diagram.

During the study period from 11 March 2020 to 31 November 2020, a total of 616 confirmed COVID‐19 patients were admitted to our COVID‐19 isolation ward. Of all of them, only 600 complete medical records can be obtained. We excluded children (27 persons), pregnant women (65 persons) and those who never had blood glucose measurements during admission (6 persons). Therefore, a total of 506 participants were included in this study (Figure 1). Meanwhile, for the analysis of intensive care, 242 subjects were excluded because they went into intensive care unit since the first admitted to hospital, so that 264 subjects met the requirement for the analysis of intensive care.

The characteristic of the participants is presented in Table 1. Diabetes was found in 28.46% of individuals. Most of the subjects in this study were below 60 years old, with a median (IQR) age 51 (22) years old. Females are more prevalent compare to males (56.32% vs. 43.68%). Despite diabetes, the most common comorbidity found in this study was hypertension (30.80%) and chronic kidney disease (24.20%). The proportion of severity levels among subjects at the admission time was as follows; mild 11.07%, moderate 34.39%, severe 8.89% and critical 45.85% (ARDS 44.66%, sepsis 1.19%, septic shock 6.72%). The mean hospital duration in this study was 13 (12 days).

**TABLE 1:** Characteristics of subjects.

Variable	Total	Diabetes	
		Yes	No
		144 (28.46)	362 (71.54)
Age, years old—Median (IQR)	51 (22)	57 (15)	48.5 (22)
Age category			
≥60 years old	143 (28.26)	60 (41.67)	83 (22.93)
<60 years old	363 (71.47)	84 (58.33)	279 (77.07)
Sex			
Male—*n* (%)	221 (43.68)	67 (46.53)	154 (42.54)
Female—*n* (%)	285 (56.32)	77 (53.47)	208 (57.46)
Comorbidity			
Hypertension—*n* (%)	154 (30.80)	60 (42.25)	94 (26.6)
Heart disease—*n* (%)	57 (11.40)	22 (15.49)	35 (9.78)
Chronic obstructive pulmonary disease—*n* (%)	23 (4.60)	6 (4.23)	17 (4.75)
Stroke—*n* (%)	21 (4.20)	5 (3.52)	16 (4.47)
Chronic kidney disease—*n* (%)	121 (24.20)	43 (30.28)	78 (21.79)
Liver disease—*n* (%)	5 (0.99)	1 (0.69)	4 (1.10)
Other immunodeficiency—*n* (%)	19 (3.80)	8 (5.64)	11 (3.08)
Laboratory parameters			
RBGs, mg/dL—median (IQR)	109 (54)	139.5 (122)	105 (41)
CRP, mg/dL—median (IQR)	4.5 (10.9)	6.5 (13.5)	3.97 (9.2)
Feritin, ng/mL—median (IQR)	542.5 (1172)	772.5 (1235.52)	474 (1043)
D‐dimer, ng/mL—median (IQR)	1570 (2260)	2260 (3315.8)	1390 (2390.42)
LDH, u/L—median (IQR)	485.49 (322)	523.5 (378.5)	477.14 (306.99)
NLR—median (IQR)	6.42 (5.41)	6.38 (5.45)	6.51 (5.45)
MLR—median (IQR)	0.49 (0.34)	0.45 (0.272)	0.5 (0.363)
Hospital duration, day—median (IQR)	13 (12)	15 (16)	13 (8)
Severity level			
Mild—*n* (%)	56 (11.07)	6 (4.17)	50 (13.81)
Moderate—*n* (%)	174 (34.39)	32 (22.22)	142 (39.23)
Severe—*n* (%)	44 (8.89)	14 (9.72)	30 (8.29)
Critical—*n* (%)	232 (45.85)	92 (63.89)	140 (38.67)
ARDS—*n* (%)	226 (44.66)	91 (63.19)	135 (37.29)
Sepsis—*n* (%)	6 (1.19)	3 (2.08)	3 (0.83)
Septic shock—*n* (%)	34 (6.72)	17 (11.81)	17 (0.83)

### 3.2 The association between diabetes and outcome

Of all of the subjects included in COVID‐19 isolation ward, 297 (58.70%) subjects were admitted to the intensive care unit, while 140 (27.67%) subjects were dead during admission. Among subjects with diabetes versus subjects without diabetes, a higher proportion was admitted to intensive care (86.11% vs. 47.79%, respectively *p* < .001, Table S2). The proportion of DM patients undergoing intensive care was 38.8%, while the non DM group was 10.055%, bivariate analysis obtained an RR of 3.27 (95% CI: 2.12–5.03, Table S2). The proportion of DM patients who died during COVID‐19 treatment was higher than non‐diabetic (45.14% vs. 20.72%, respectively *p* < .001, Table S2), bivariate analysis obtained an RR 2.18 (95% CI: CI 1.66–2.86, Table S3).

**FIGURE 2:**
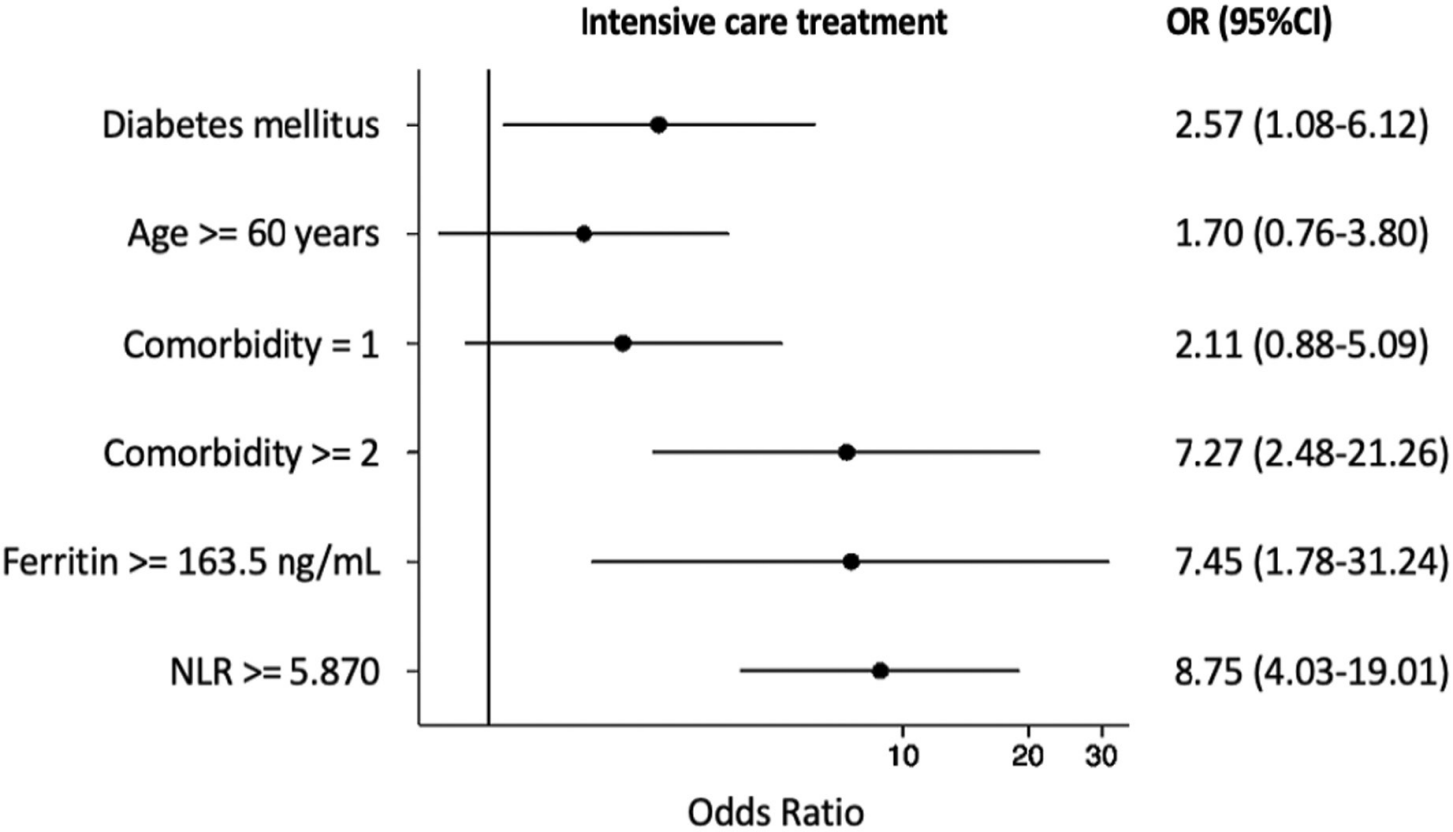
The association between diabetes and intensive care treatment in COVID‐19 patients.

**FIGURE 3:**
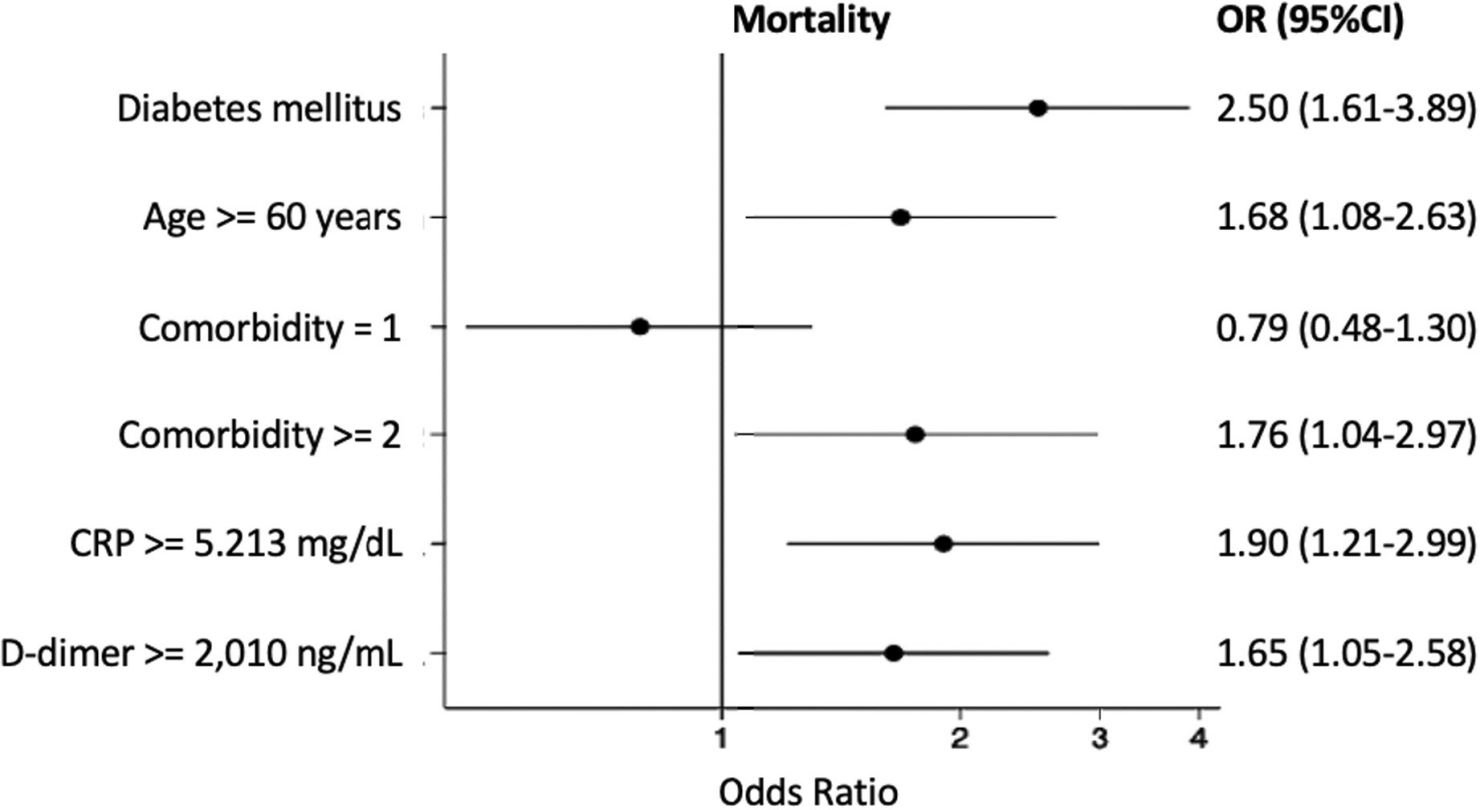
The association between diabetes and mortality in COVID‐19 patients.

In multivariate logistic regression analysis, diabetes was associated with increased odds of admitted on intensive care OR 2.57 (95% CI: 3.52–10.43) (Figure 2), and death OR 2.50 (95% CI: 1.61–3.89) (Figure 3) after adjustment with age, sex, comorbidity, random blood glucose levels, C‐reactive protein, ferritin, d‐dimer, lactate dehydrogenase levels, neutrophile and monocyte to lymphocyte ratio levels.

### 3.3 Laboratory parameters as a mortality predictor in diabetes subjects

The accuracy of laboratory parameters to predict mortality in diabetes subjects was shown based on the area under the curve (AUC) of each parameter. All of the laboratory parameters had AUC below 0.70 in predicting mortality of diabetes subjects except for ferritin (AUC: 0.71, 95% CI: 0.62–0.79) and LDH (AUC: 0.70, 95% CI: 0.61–0.78). The optimal cut‐off for ferritin to predict mortality among subjects with diabetes was 786 ng/mL (sensitivity: 0.72, specificity: 0.68) and the optimal cut off for LDH was 514.94 u/L (sensitivity: 0.71, specificity: 0.62) (Table S1).

### 3.4 Factors associated with mortality among diabetes subjects

We explore factors affecting mortality in diabetes subjects. Based on bivariate analysis, some variables have a statistically significant association with mortality. They were random blood glucose levels, CRP, ferritin, LDH, D‐dimer and ARDS (Table S3). The multivariate analysis was performed. Based on multivariate analysis, it is seen that age above 70 years old, RBGs level above 250 mg/dL or below 140 mg/dL, ferritin level above 786 ng/mL and presence of ARDS increased the odds of mortality among subjects with diabetes. Meanwhile, comorbidity, NLR above 4.69 and MLR above 0.8 are confounding factors that attenuate the other risks (Table 2).

**TABLE 2:** Risk factor of mortality among COVID‐19 with diabetes subjects.

Variable	Coefficient B	*p*‐Value	OR	95% CI
RBGs mg/dL				
<140	1618	.003	5.04	1.72–14.78
>250	1768	.006	5.86	1.65–20.78
Age ≥ 70 years old	.283	.033	1.33	1.02–1.72
NLR ≥ 4.69	.660	.130	1.94	0.82–4.55
Ferritin ≥786 ng/mL	1731	.000	5.65	2.40–13.29
ARDS	1198	.009	3.31	1.35–8.11
Constanta	−3498	.000	0.04	0.01–0.12

## 4 DISCUSSION

Our study is different from previous studies in that we not only assessed the outcome between DM and COVID‐19, but we also assessed the laboratory parameter for predicting the outcome between DM and COVID‐19. In our study, we found that the proportion of DM among COVID‐19 confirmed patients admitted to our hospital was 28.5%. Furthermore, DM increased the risk of having intensive care treatment by 2.57, and death by 2.50 compared to those who did not have DM. This finding is consistence with other findings including a meta‐analysis who included 76 studies, in which 10 studies (for severe outcome) and 8 studies (for mortality outcome) came from outside China. The risk of having severe condition increased by 2.02 (95 CI: 1.44–2.84, *I* ^2^: 55%, *p* < .02) and the risk of having mortality increased by 2.21 (95 CI: 1.83–2.66, *I* ^2^: 50%, *p* < .01) in DM patients compared to those who did not had DM outside China.^21^ Other meta‐analysis including in hospital adults' patients with COVID‐19 found the same. The risk of in hospital mortality increased by 2.39 (95 CI: 1.65–3.46, *I* ^2^: 62%, *p*: .001).^22^ However, those meta‐analysis failed to prove diabetes increased the risk of severe conditions.^22^ Other meta‐analysis including three study analysed intensive care treatment among COVID‐19 also failed to prove diabetes increased the risk of having intensive care treatment. (OR: 1.47, 95 CI: 0.38–5.67, *I* ^2^: 63%, *p*: .07).^23^

The results of previous meta‐analysis studies are still conflicting and had high heterogeneity. In this study, the OR of the relationship between diabetes and intensive care was high, 2.57. However, if we looked at the confidence interval in this relationship (3.52–10.43), the confidence interval is wide. Some reasons can explain this finding. First, besides clinical consideration, physicians' decision to admit subjects to an intensive care facility was affected by beds availability and family consent. Also, a small sample size might give a wide range of confidence intervals. From all 606 subjects admitted to our hospital, we could assess only 264 subjects (42.86%) after the exclusion. Lastly, we used OR rather than risk ratio (RR) as an association measurement because we used logistic regression for multivariate analysis. Therefore, we can assume that this relationship is not strong and is most likely affected by chance. Further research with clinically objective outcomes like ARDS, sepsis or other critical conditions is recommended.

Besides of COVID‐19, it is well known that subjects with diabetes are prone to have infections compared to non‐diabetes subjects. There is also paucity evidence in epidemiological studies where DM patients had increased risk of having mortality by COVID‐19.^21^, ^22^, ^23^ The potential mechanisms that could explain the high COVID‐19 mortalities among DM subjects were impaired immune responses, glycaemic instability, chronic subclinical inflammation and the presence of associated comorbidities in diabetes subjects.^24^

Several abnormal immune system have been seen in diabetes subjects, those including higher affinity cellular binding for virus entry, inhibition of viral clearance, impaired T‐cell function, lymphopenia and exaggerated inflammatory response associated with an increased renin‐angiotensin system (RAS) activation in several tissues.^25^, ^26^ In addition, those conditions were worsened by chronic glycaemic instability and association inflammation, which contribute to increased susceptibility to hyperinflammation and cytokine storm.^26^ These findings also supported our study in which high ferritin (>786 ng/mL) increased the risk of mortality.

To date, there is no available report regarding cut‐off for laboratory findings in predicting mortality of COVID‐19 patients with diabetes. In this study, among all the laboratory parameters, only ferritin shows good performance in predicting mortality. Ferritin gives an AUC score of 0.71 with an optimal cut‐off at 786 ng/mL, giving it a sensitivity of 0.72 and specificity of 0.68. LDH seemed like a good predictor for mortality among subjects with diabetes. However, it failed to prove its association with mortality in multivariate analysis (Table S3).

Many studies have been reported that ferritin is an independent factor of poor outcomes in COVID‐19 patients.^27^ Ferritin is an intracellular protein that can store iron and plays a critical role in inflammatory diseases. It is an acute‐phase protein, which is elevated during inflammation or infection. The condition of ‘hyperferritinemia syndrome’ is proved to be associated with highly active diseases, resulting in immune activation and coagulation disturbances.^27^ Among COVID‐19 patients, subjects with diabetes conditions already had a higher baseline of ferritin.^28^, ^29^ The out‐of‐control inflammatory state in diabetes subjects who are already on impaired immune response may cause widespread tissue damage and susceptibility to poor outcomes.

Subjects with diabetes also have a greater risk for poor glycaemic control. Acute diabetes complications were likely to occur (diabetic ketoacidosis, hyperglycaemia hyperosmolar syndrome and hypoglycaemia), which then inhibits the ability to mitigate sepsis and higher mortality rate due to the complication itself.^26^ Furthermore, the presence of associated morbidities and the use of agents that can modulate angiotensin‐converting enzyme 2 (ACE2) expression have been put forth to explain the latter association.^25^ We demonstrated that age and random blood glucose levels on admission were the independent risk factors of mortality in COVID‐19 subjects with diabetes. We found age is major role when diabetes subjects reached 70 years or older. Also, subjects with RBGs levels above 250 mg/dL or below 140 mg/dL were found at increased risk of death.

Following our findings, a meta‐analysis including 22 studies showed that individuals with a more severe diabetes condition have a poorer prognosis of COVID‐19 compared to those with milder diabetes conditions.^30^ A Spanish COVID‐19 registry found admission hyperglycaemia (RBG > 180 mg/dL) regardless of diabetes diagnosis was a strong predictor of all‐cause mortality in non‐critically hospitalized patients.^31^ Moreover, the chronic hyperglycaemia condition also leads to increased fibrinogen amyloid changes, leading to hypercoagulability conditions.^32^ It is not surprising that hyperglycaemia increased the risk of poor outcomes. Current evidence showed tight glucose control with insulin infusion in ICU had a lower risk of severe symptoms and mortality. It is also well known that hypoglycaemia might produce the same effect as acute hyperglycaemia. Nevertheless, glucose variability also induced cytokines release and worsened prognosis. Currently, the optimal cut‐off for glycaemic control among COVID‐19 patients with hyperglycaemia remains elusive. It is challenging to maintain normal glycaemic in COVID‐19 patients with glycaemia, and it was even more complicated with the use of corticosteroid treatment. Therefore, increased attention is needed for blood glucose regulations in COVID‐19 patients. Further research regarding optimal glucose target and evidence of treatment is needed.

This study has several limitations to be discussed. First, we cannot analyse the time relationship in this study. That is because we did not measure the diabetes duration. Second, diabetes is a heterogeneous population. We included all subjects with diabetes conditions, including Type 1 diabetes, Type 2 diabetes, newly diagnosed diabetes, controlled or uncontrolled diabetes, those who received insulin or oral diabetes medication. In addition, there were several variables that we were not assessed in this study such as smoking habits, nutritional status, level of glycaemic control (HbA1c) at admission and glucose control at admission. Therefore, there is no specificity in this study, nor we performed a sub‐analysis of which of these different factors might play a role in the outcome. Lastly, our study is conducted in a tertiary facility and one of the largest hospitals in Indonesia that the government‐appointed as an advanced referral hospital. About 45.85% of subjects in this study already came with critical conditions, and 8.89% were already in severe conditions. Therefore, readers should be careful in generalizing our results.

In accordance with our findings, a meta‐analysis including 22 studies showed individuals with a more severe diabetes condition have a poorer prognosis of COVID‐19 compared to those with milder diabetes conditions.^30^ A Spanish COVID‐19 registry found admission hyperglycaemia (RBG > 180 mg/dL) regardless of diabetes diagnosis was a strong predictor of all‐cause mortality in non‐critically hospitalized patients.^31^ Moreover, the chronic hyperglycaemia condition also leads to increased fibrinogen amyloid changes, leading to hypercoagulability conditions.^32^ It is not surprising that hyperglycaemia increased the risk of poor outcome.^33^ Also, hypoglycaemia might produce the same effect as acute hyperglycaemia. The optimal cut‐off for COVID‐19 patients with hyperglycaemia treatment remains elusive. Current evidence showed tight glucose control with insulin infusion in ICU had a lower risk of severe symptoms and mortality.^34^ But glucose variability also induced cytokines release and worsen prognosis.^34^ It is challenging to maintain normal glycaemic in COVID‐19 patients with glycaemia and it was even harder with the use of corticosteroid treatment. Increased attention is needed for blood glucose regulations in COVID‐19 patients. Further research regarding optimal glucose target and evidence of treatment is needed.

## 5 CONCLUSION

Diabetes mellitus increases risk intensive care admission and in hospital mortality in COVID‐19. Multivariate analysis showed that older age, RBG on admission, high ferritin level, and presence of ARDS increased the odds of mortality among individuals with diabetes.

## AUTHOR CONTRIBUTIONS

**Md Ikhsan Mokoagow:** Conceptualization (lead); data curation (equal); formal analysis (equal); investigation (equal); methodology (equal); project administration (equal); writing – original draft (equal). **Dante Saksono Harbuwono:** Data curation (equal); formal analysis (equal); validation (equal). **Ida Ayu Kshanti:** Data curation (equal); formal analysis (equal); validation (equal). **C. Martin Rumende:** Data curation (equal); formal analysis (equal); validation (equal). **Imam Subekti:** Validation (equal); writing – review and editing (equal). **Kuntjoro Harimurti:** Validation (equal); writing – review and editing (equal). **Khie Chen:** Validation (equal); writing – review and editing (equal). **Hamzah Shatri:** Validation (equal); writing – review and editing (equal).

## CONFLICT OF INTEREST STATEMENT

The authors declare no conflicts of interest.

## Supporting information


Table S1.

## Data Availability

Data sharing is not applicable to this article as no new data were created or analyzed in this study.
