# Supplementary material for: Increased risk of poor clinical outcome in COVID‐19 patients with diabetes mellitus and in‐hospital mortality predictors: A retrospective cohort from a tertiary hospital in Indonesia
Source: Endocrinol Diabetes Metab. 2023 Oct 9;6(6):e454. doi: 10.1002/edm2.454 (PMC10638617; doi:10.1002/edm2.454)
Supplement: Supplementary file 1 — Table S1. [file EDM2-6-e454-s001.docx]

**Supp 1**. Laboratory parameters as a mortality predictor in COVID-19 with diabetes subjects

| Laboratory parameter | *AUC* | *Cut-off** | Spec* | Sens | Spec | PPV | NPV | LR+ | LR- |
| --- | --- | --- | --- | --- | --- | --- | --- | --- | --- |
| CRP (mg/dL) | 0.650 | > 5,213 | 0,710 | 0,621 | 0,601 | 0,373 | 0,806 | 1,556 | 0,631 |
| LDH (u/L) | 0.675 | > 353,5 | 0,890 | 0,871 | 0,262 | 0,311 | 0,842 | 1,180 | 0,492 |
| Ferritin (ng/mL) | 0.662 | > 304,3 | 0,706 | 0,779 | 0,352 | 0,315 | 0,806 | 1,202 | 0,628 |
| d-dimer (ng/mL) | 0.645 | > 2.010 | 0,740 | 0,564 | 0,637 | 0,373 | 0,793 | 1,554 | 0,684 |
| NLR | 0.500 | > 8,085 | 0,848 | 0,221 | 0,762 | 0,263 | 0,719 | 0,929 | 1,022 |
| MLR | 0.503 | > 0,364 | 0,800 | 0,643 | 0,383 | 0,287 | 0,740 | 1,042 | 0,932 |
| RBGs (mg/dL) | 0.557 | > 180 | - | 0,271 | 0,861 | 0,427 | 0,755 | 1,950 | 0,847 |

**Supp *2*.** The Association between Diabetes and Intensive care treatment in COVID-19 patients

| Variable | | Intensive care treatment-n(%) | | *RR crude*  (95%CI) | *OR adjusted*  (95%CI) |
| --- | --- | --- | --- | --- | --- |
|  |  | Yes  N = 55 (20.83%) | No  N = 209 (79.17%) |  |  |
| Diabetes | |  |  |  |  |
|  | **Yes** | **21 (38.18%)** | **21 (10.055)** | **3.27 (2.12-5.03)**** | **2.57 (1.08-6.12)*** |
|  | **No** | **34 (61.82%)** | **188 (89.95%)** | **1.00** | **1.00** |
| Age, years | |  |  |  |  |
|  | > 60 years | 26 (47.27%) | 34 (16.27%) | 3.05 (1.96-4.75)** | 1.70 (0.76-3.80) |
|  | < 60 years | 29 (52.73%) | 175 (83.73%) | 1.00 | 1.00 |
| Sex | |  |  |  |  |
|  | Male | 27 (49.09%) | 72 (34.45%) | 1.61 (1.00-2.56)* |  |
|  | Female | 28 (50.91%) | 137 (65.55%) | 1.00 |  |
| Comorbidity | |  |  |  |  |
|  | > 2 comorbids | 19 (34.55%) | 15 (7.18%) | 5.24 (2.59-10.62)** | 7.27 (2.48-21.26)** |
|  | 1 comorbid | 23 (41.82%) | 85 (40.67%) | 2.00 (1.01-3.95)* | 2.11 (0.88-5.09) |
|  | Without comorbid | 13 (23.64%) | 109 (52.15%) | 1.00 | 1.00 |
| RBG, mg/dL | |  |  |  |  |
|  | > 180 mg/dL | 15 (27.27%) | 17 (8.13%) | 2.72 (1.71-4.33)** |  |
|  | < 180 mg/dL | 40 (72.73%) | 192 (91.87%) | 1.00 |  |
| CRP, mg/dL | |  |  |  |  |
|  | > 2,587 mg/dL | 43 (78.18%) | 86 (41.15%) | 3.75 (2.07-6.78)** |  |
|  | < 2,587 mg/dL | 12 (21.82%) | 123 (58.85%) | 1.00 |  |
| Ferritin, ng/mL | |  |  |  |  |
|  | > 163,5 ng/mL | 52 (94.55%) | 130 (62.20%) | 7.81 (2.51-24.28)** | 7.45 (1.78-31.24)** |
|  | < 163,5 ng/mL | 3 (5.45%) | 79 (37.80%) | 1.00 | 1.00 |
| D-dimer, ng/mL | |  |  |  |  |
|  | > 565 ng/mL | 50 (90.91%) | 113 (54.07%) | 6.20 (2.56-15.02)** |  |
|  | < 565 ng/mL | 5 (9.09%) | 96 (45.93%) | 1.00 |  |
| LDH, u/L | |  |  |  |  |
|  | > 277 u/L | 53 (96.36%) | 179 (85.65%) | 3.66 (0.94-14.28)* |  |
|  | < 277 u/L | 2 (3.64%) | 30 (14.35%) | 1.00 |  |
| NLR | |  |  |  |  |
|  | > 5,870 | 39 (70.91%) | 41 (19.62%) | 5.61 (3.34-9.42)** | 8.75 (4.03-19.01)** |
|  | <5,870 | 16 (29.09%) | 168 (80.38%) | 1.00 | 1.00 |
| MLR | |  |  |  |  |
|  | > 0,300 | 46 (83.64%) | 109 (52.15%) | 3.59 (1.84-7.03)** |  |
|  | < 0,300 | 9 (16.36%) | 100 (47.85%) | 1.00 |  |

**Supp *3*.** The Association between Diabetes and mortality in COVID-19 patients

| Variable | | Mortality– n(%) | | *RR crude*  (95%CI) | *OR adjusted*  (95%CI) |
| --- | --- | --- | --- | --- | --- |
|  |  | Yes  N = 140 (27.67%) | No  N = 366 (72.33%) |  |  |
| Diabetes | |  |  |  |  |
|  | **Yes** | **65 (45,14)** | **79 (54,86)** | **2.18 (1.66-2.86) **** | **2,50 (1.61-3,89) **** |
|  | **No** | **75 (20,72)** | **287 (79,28)** | **1,00** | **1,00** |
| Age, years | |  |  |  |  |
|  | > 60 years | 54 (37,76) | 89 (62,24) | 1.59 (1.21-2.19) ** | 1,68 (1,08-2,63) * |
|  | < 60 years | 86 (23,69) | 277 (76,31) | 1,00 | 1,00 |
| Sex | |  |  |  |  |
|  | Male | 71 (32,13) | 150 (67,87) | 1,32 (0.99-1.74) |  |
|  | Female | 69 (24,21) | 216 (75,79) | 1,00 |  |
| Comorbidity | |  |  |  |  |
|  | > 2 comorbid | 44 (42,31) | 60 (57,69) | 3.23 (2.03-5.14) ** | 1,76 (1,04-2,97) * |
|  | 1 comorbid | 41 (24,70) | 125 (75,30) | 1.95 (1.23-3.10) | 0,79 (0,48-1,30) |
|  | Without comorbid | 55 (23,31) | 181 (76,69) | 1,00 | 1,00 |
| RBG, mg/dL | |  |  |  |  |
|  | > 180 mg/dL | 38 (42,70) | 51 (57,30) | 1.96 (1.48-2.59) ** |  |
|  | < 180 mg/dL | 102 (24,46) | 315 (75,54) | 1,00 |  |
| CRP, mg/dL | |  |  |  |  |
|  | > 5,213 mg/dL | 87 (37,34) | 146 (62,66) | 3.76 (2.61-5.40) ** | 1,90 (1,21-2,99) ** |
|  | < 5,213 mg/dL | 53 (19,41) | 220 (80,59) | 1,00 | 1,00 |
| Ferritin, ng/mL | |  |  |  |  |
|  | > 304,3 ng/mL | 109 (31,50) | 237 (68,50) | 1,91 (1,20-3,18) ** |  |
|  | < 304,3 ng/mL | 31 (19,38) | 129 (80,62) | 1,00 |  |
| d-dimer, ng/mL | | | | | |
|  | > 2.010 ng/mL | 79 (37,26) | 133 (62,74) | 3.89 (2.27-6,65)** | 1,65 (1,05-2,58) * |
|  | < 2.010 ng/mL | 61 (20,75) | 233 (79,25) | 1,00 | 1,00 |
| LDH, u/L | |  |  |  |  |
|  | > 353,5 u/L | 122 (31,12) | 270 (68,88) | 3.06 (2.27-4.12) ** |  |
|  | < 353,5 u/L | 18 (15,79) | 96 (84,21) | 1,00 |  |
| NLR | |  |  |  |  |
|  | > 8,085 | 31 (26,27) | 87 (73,73) | 3,27 (2.46-4.36)** |  |
|  | < 8,085 | 109 (28,09) | 279 (71,91) | 1,00 |  |
| MLR | |  |  |  |  |
|  | > 0,364 | 90 (28,66) | 224 (71,34) | 2,78 (1,93-3.99)** |  |
|  | < 0,364 | 50 (26,04) | 142 (73,96) | 1,00 |  |
